# Binding of EphrinA5 to RET receptor tyrosine kinase: An *in vitro* study

**DOI:** 10.1371/journal.pone.0198291

**Published:** 2018-06-11

**Authors:** Yixin Liu, Heidi Kaljunen, Ana Pavić, Tuulia Saarenpää, Juha P. Himanen, Dimitar B. Nikolov, Adrian Goldman

**Affiliations:** 1 Molecular and Integrative Biosciences, Faculty of Biological and Environmental Sciences, University of Helsinki, Helsinki, Finland; 2 Structural Biology Program, Memorial Sloan-Kettering Cancer Center, New York, New York, United States; 3 Astbury Centre for Structural Molecular Biology, School of Biomedical Sciences, Faculty of Biological Sciences, University of Leeds, Leeds, United Kingdom; Hungarian Academy of Sciences, HUNGARY

## Abstract

Eph/Ephrin signaling pathways are crucial in regulating a large variety of physiological processes during development, such as cell morphology, proliferation, migration and axonal guidance. EphrinA (efn-A) ligands, in particular, can be activated by EphA receptors at cell-cell interfaces and have been proposed to cause reverse signaling *via* RET receptor tyrosine kinase. Such association has been reported to mediate spinal motor axon navigation, but conservation of the interactive signaling pathway and the molecular mechanism of the interaction are unclear. Here, we found *Danio rerio* efn-A5b bound to *Mus musculus* EphA4 with high affinity, revealing structurally and functionally conserved EphA/efn-A signaling. Interestingly, we observed no interaction between efn-A5b and RET from zebrafish, unlike earlier cell-based assays. Their lack of association indicates how complex efn-A signaling is and suggests that there may be other molecules involved in efn-A5-induced RET signaling.

## Introduction

Motor neurons in the lateral and median divisions of the lateral motor neuron innervate dorsal and ventral limbs. The right connection of neurons is guided by correct positioning of axons and dendrites. A small number of receptor proteins in conjunction with extracellular cues direct the positioning of axons during embryonic development. Hence, the cues must be highly precise and versatile in order to assemble the complex nervous wiring system [1]. Multiple molecules are involved in axonal guidance, of which Eph receptor tyrosine kinases and their ephrin ligands are prominent [2–4].

The Eph receptors consist of two large subfamilies, EphAs and EphBs, classified by their sequence identity as well as their preference for binding to either glycophosphatidylinositol (GPI)-anchored ephrinA ligands or transmembrane ephrinB ligands [5]. Typically, EphAs (EphA1-A10) selectively interact *in trans* with ephrinA ligands (efn-A1-A6) while six classes of EphBs bind to three ephrinBs (efn-Bs), although exceptions have been reported (EphA4 [5] and efn-A5 [6] cross-interact with members of the other subfamily). One special feature of Eph/ephrin molecules is their complex signaling modes: they transduce bidirectional signals based on their distribution on the communicating cell surfaces [7,8]. Because efn-As are GPI-anchored membrane proteins, they have to induce reverse signaling by recruiting other transmembrane receptors into the signaling-competent cluster. Research has been undertaken to identify co-receptors that regulate efn-A reverse signaling [9–12]. Recently, mouse RET receptor tyrosine kinase has been reported to bind to mouse efn-A5 (mefn-A5) and mouse efn-A2 (mefn-A2). mRET/mefnA signaling mediates axonal growth dorsally in the presence of glial cell-derived neurotrophic factor (GDNF) and mEphA4 or mEphA7 receptor *in vivo* [13,14].

RET receptor tyrosine kinase is a single-span membrane protein, encoded by an oncogene initially discovered in 1985 [15]. It is well known to primarily signal upon binding to its ligand GDNF, a soluble trophic factor of the GDNF family ligands (GFLs), only in the presence of GDNF family receptor αs (GFRαs) [16]. The extracellular domain of RET is responsible for ligand recognition and consists of four cadherin-like domains (CLDs) as well as a cysteine-rich domain (CRD) before the single transmembrane helix. Signaling of GDNF/GFRα1/RET is important for motor neuron maintenance and neurite outgrowth [17,18]. Interestingly, efn-A5 was found to potentially compete with GFRα1 to bind with RET [13] and mefn-A5 was shown to involve an interaction with the cadherin Celsr3, Frizzled3 (Fzd3), mRET and mGFRα1 [14], suggesting a complex but selective cross talk between the two conventional efn-A/EphA and GDNF/GFRα1/RET pathways.

Eph, efn, RET, GDNF and GFRα are all highly conserved during evolution [19–23]. Especially for efn-As, they have an overall sequence similarity as high as 80% among their orthologs (S1 Fig). Our recent study found that zebrafish GDNF/GFRα1 could activate human RET, revealing the structural and functional conservation of RET from an evolutionary perspective [24]. Because mammalian RETs are prone to misfold [25,26] and thus difficult to produce, we wanted to determine if the binding of RET to efn-A5 is conserved in lower vertebrates, for example zebrafish, and characterize their binding behavior to elucidate the underlying molecular mechanism of the reverse signaling of efn-A5. In this work, we therefore investigated the interaction between zebrafish efn-A5b isoform (zefn-A5) and zebrafish RET (zRET) *in vitro* to explore the binding between the two proteins. We produced extracellular domains (ECD) of zRET, zefn-A5, zGDNF and zGFRα1 in insect cells and assessed their association by a combination of qualitative and quantitative methods. Here, we show monomeric zefn-A5^ECD^ and zRET^ECD^ purified from insect cells do not directly interact with each other *in vitro*, while monomeric zefn-A5^ECD^ exhibits high affinity to the dimeric ligand-binding domain (LBD) of mouse EphA4, similar to its mammalian orthologs. Our findings suggest that the reverse signaling of efn-As may be far more complex than previously indicated.

## Materials and methods

### DNA constructs

zefn-A5^ECD^ (residues 21–204, NCBI reference sequence NP_571101.1) and zefn-A2^ECD^ (residues 17–174, NP_571097.1) were fused into a modified FastBac vector with N-terminal Flag-His_8_-tags and a thrombin cleavage site (referred to as pK503.9 vector) [27]. Preparation of plasmids containing zRET^ECD^ (residues 22–626, NP_858048.2), zGFRα1^ECD^ (residues 31–351, NP_571805.1) and mature zGDNF (residues 90–236, NP_571807.1) in pK503.9 vector has been described previously [24]. mEphA4^LBD^ (residues 29–210, NP_031962.2) was cloned into pMA152a vector attached to a C-terminal human lgG1 hinge region and Fc tag, referred to as mEphA4^LBD^. mEphA4^ECD^ (residues 27–548, NP_031962.2) with a C-terminal His tag was cloned into pPICZαA vector. Herein, zefn-A5^ECD^, zefn-A2^ECD^, zRET^ECD^ and zGFRα1^ECD^ refer to Flag-His_8_-tagged proteins and zGDNF refers to zGDNF with a N-terminal Flag tag.

### Cell culture and protein expression

*Spodoptera frugiperda* (Sf9) and *Trichoplusia ni* High Five (Hi5) insect cells (Thermo Fisher Scientific) were maintained below 2 million cells/ml in suspension at 27˚ C. Recombinant baculovirus bacmid DNA was generated using X-treme gene transfection reagent according to the manufacturer’s protocol (Roche). Initial (passage zero, V0) recombinant baculovirus was harvested 60-hour post infection in Sf9 cells and virus was amplified up to passage two (V2) [24]. Baculovirus-infected insect cell (BIIC) stocks were prepared as described [28] for large-scale expression. Protein expression for secreted proteins continued for 72 hours after BIIC infection at 27˚ C using Hi5 cells. mEphA4^ECD^ construct was transformed into *Pichia pastoris*. The selection of multi-copy expression stain was achieved by using a high concentration of zeocin at 100 μg/ml and the selected stain was used for large-scale culture. In brief, 25 ml starter culture was inoculated and grown at 30˚ C. Large-scale culture was prepared by transferring 10–20 ml starter culture to 1 L Buffered Glycerol-complex Medium (BMGY) culture 24-hour post induction. Cells were grown at 30˚ C until an OD_600_ = 2–6 and were afterwards harvested by centrifugation at room temperature (RT). Cell pellets were resuspended in Buffered Methanol-complex Medium (BMMY) to give a final OD_600_ = 1. Cells were cultured in baffled flasks at 28˚ C with shaking at 280 rpm and protein expression was allowed for 72–84 hours post induction. Methanol (5% v/v) was supplemented every 24 hours and aliquots were taken to monitor protein expression.

### Protein purification

Recombinant proteins (zefn-A5^ECD^, zefn-A2^ECD^, zRET^ECD^, zGFRα1^ECD^, and zGDNF) were secreted into the medium and harvested by centrifugation as previously described [24]. The protein-containing medium was concentrated and buffer-exchanged into binding buffer (20 mM Tris, 300 mM NaCl, 10 mM imidazole, pH 7.5) using a Pellicon concentrator (Millipore EMD) with a molecular weight (MW) cut-off of 5 kDa (for zefn-A5^ECD^, zefn-A2^ECD^ and zGDNF) or 30 kDa (for zRET^ECD^ and zGFRα1^ECD^). For zefn-A5^ECD^, zefn-A2^ECD^, zRET^ECD^ and zGFRα1^ECD^ purification, we adopted a two-step purification method: tagged proteins were first selected using a Ni-affinity gravity column (QIAGEN) and secondly purified using anti-Flag resin (Biotool, Bimake) and eluted with 300 μg/ml (Biotool, Bimake) poly-Flag peptide solution. Proteins were concentrated to 500 μl with Amicon centrifuge concentrators (Millipore EMD) and further purified by size-exclusion chromatography (SEC) using an Äkta purifier (GE Healthcare) on a Superdex 200 GL 10/300 column (GE Healthcare) pre-equilibrated in SEC buffer (20 mM HEPES, 150 mM NaCl, pH 8). Purification of zGDNF was performed as previously described [24]. mEphA4^LBD^ was expressed using Hi5 cells and purified by affinity chromatography using Fast Flow Protein-A Sepharose (GE Healthcare). mEphA4^ECD^ was expressed and secreted using *Pichia pastoris*. His-tagged recombinant protein was purified using a 5-ml His-Trap HP column (Amersham-Pharmacia) and was further polished by SEC on a Superdex-200 10/30 column (GE Healthcare). Protein concentration was determined from the measured absorbance at 280 nm wavelength (NanoDrop Spectrophotometer ND-1000, Thermo Fisher Scientific) using extinction coefficient and MW calculated by ExPASy ProtParam tool for each non-glycosylated protein. The concentration and molarity of zGDNF and mEphA4^LBD^ were calculated for their monomeric forms and they were calculated the same way when used in all the assays.

### One-dimensional native polyacrylamide gel electrophoresis (PAGE)

Purified recombinant proteins were analyzed using native PAGE. zefn-A5^ECD^ was kept at 8 μM and the amount of other proteins were calculated based on theoretical molecular weights without glycosylation resulting in a molar ratio of 2:1:1:1:1 (zefn-A5^ECD^ (24.6 kDa): zRET^ECD^ (72.9 kDa): zGDNF (18.1 kDa): zGFRα1^ECD^ (40.3 kDa): mEphA4^LBD^ (49.8 kDa)). Samples were mixed and incubated at room temperature for 30 min before adding sample buffers. Procedures for 1-D Native PAGE, including both Blue Native (BN) PAGE and Clear Native (CN) PAGE, were slightly modified and adapted to our electrophoresis system for Mini-PROTEAN Precast Gels (Bio-Rad) based on the protocol previously described [29]. Electrophoresis was performed at 4˚ C with constant voltage at 100 V for 2–6 hours. For BN PAGE, protein complexes and individual proteins were resolved on 4–20% gradient gels while 7.5% gels were used for CN PAGE due to the resolution limit of CN PAGE under the these running conditions. Afterwards, gels were analyzed by Western blot with goat anti-Human IgG Fc (HRP) (ab97225, Abcam) antibody and imaged using a ChemiDoc XRS+ System (Bio-Rad) to detect bound antibodies. Experiments were performed in triplicate.

### Peptide extraction and mass spectrometry

After proteins were resolved on a 4–20% gradient gel, the protein bands of interest were excised for mass spectrometry (MS) analysis. Peptides were extracted from the gel slices by in-gel digestion according to the methods previously described [30]. Cysteine bonds were reduced with 0.045 M dithiothreitol (#D0632 Sigma-Aldrich, USA) for 20 min at 37˚ C and alkylated with 0.1 M iodoacetamide (#57670 Fluka, Sigma-Aldrich, USA) at RT. Samples were digested by adding 0.75 μg trypsin (Sequencing Grade Modified Trypsin, V5111, Promega) and incubating overnight at 37˚ C. After digestion peptides were purified with C18 microspin columns (Harvard Apparatus) according to manufacturer’s protocol. The dried peptides were reconstituted in 30 μl buffer A containing 0.1% trifluoroacetic acid (TFA) in 1% acetonitrile (ACN).

Liquid chromatography coupled to tandem mass spectrometry (LC-MS/MS) analysis was carried out on an EASY-nLC1000 (Thermo Fisher Scientific) connected to a Velos Pro-Orbitrap Elite hybrid mass spectrometer (Thermo Fisher Scientific) with nano electrospray ion source (Thermo Fisher Scientific). The LC-MS/MS samples were separated using a two-column setup consisting of a 2 cm C18-Pepmap trap column (Thermo Fisher Scientific), followed by 15 cm C18-Pepmap analytical column (Thermo Fisher Scientific). The linear separation gradient consisted of 5% buffer B for 5 min, 35% buffer B for 60 min, 80% buffer B for 5 min and 100% buffer B for 10 min at a flow rate of 0.3 μl/min (buffer B: 0.1% TFA acid in 98% acetonitrile). 6 μl of sample was injected per LC-MS/MS run and analyzed. Full MS scan was acquired with a resolution of 60000 at normal mass range in the Orbitrap analyzer and followed by collision-induced dissociation (CID) tandem MS (MS2) ion trap scans of top 20 most intense precursor ions (energy 35). Data were acquired using LTQ Tune software. The MS2 scans were searched against homemade protein database including three protein sequences of zRET^ECD^, zGFRα1^ECD^ and zGDNF of our constructs described above using the SEQUEST search algorithms in Thermo Proteome Discoverer. The allowed mass error for the precursor ions was 15 ppm and for the fragment ions was as 0.8 Da. A static residue modification parameter was set for carbamidomethyl +57021 Da (C) of cysteine residue. Methionine oxidation was set as dynamic modification +15995 Da (M). Only full-tryptic peptides were allowed and a maximum of one missed cleavage was considered.

### Bio-layer interferometry technology system (BLItz)

Binding kinetics for zefn-A5^ECD^/zRET^ECD^ and zefn-A5^ECD^/mEphA4^LBD^ association were measured using BLItz system with Ni-NTA biosensors (ForteBio Inc.). Sensor tips were pre-hydrated for 10 min in SEC buffer supplemented with 0.5% Tween-20. zefn-A5^ECD^ at a concentration of 140 μM or zRET^ECD^ at a concentration of 110 μM was immobilized to Ni-NTA sensor tips for 3 min, during which the binding of bait proteins reached saturation. Subsequent association of prey proteins to the baits was allowed for 2 min followed by a 3-min dissociation step. As a positive control, a concentration series of 0.01, 0.04, 0.07, 0.14, 0.35, 0.70, 3.50 and 17.5 μM of mEphA4^LBD^ was used to interact with immobilized zefn-A5^ECD^. We tested 110 μM untagged zRET^ECD^ as analyte to interact with immobilized zefn-A5^ECD^ and the experiment was performed three times independently. When zRET^ECD^ was immobilized, 104 and 160 μM untagged zefn-A5^ECD^ was used as prey. Low bulk shift at the beginning of the association and dissociation phases caused by slight changes in buffer was compensated by using buffer as a reference sample to reduce the background. Assays were performed according to the instrument manual. Data were exported from BLItz pro software and replotted with Graphpad Prism 6. Plateau values of binding as reflected by changes in optical thickness (nm) were used to calculate the *K*_d_-value using nonlinear curve fitting with one binding site (total binding model).

### Pull down assays

#### Anti-Flag resin pull down

zefn-A5^ECD^ was immobilized to anti-Flag resin as bait. Protein complexes were prepared by mixing zefn-A5^ECD^/zRET^ECD^ (tag-cleaved) or zefn-A5^ECD^/mEphA4^LBD^ with a molar ratio of 2:1 and incubating this mixture at RT for 30 min. The concentration of zefn-A5^ECD^ was kept at 8 μM in the final reaction mixtures. 5 μl of pre-washed anti-Flag resin was then added to the samples coupled with 400 μl binding buffer (SEC buffer with 0.5% Tween-20). After one-hour spin mixing at 4˚ C, beads were pelleted by centrifugation and washed three times with binding buffer. Proteins bound to the beads were eluted with 300 μg/ml poly-Flag peptide or 100 mM glycine, pH 2.9. Supernatant containing eluted proteins was collected and examined using SDS-PAGE.

#### Protein G bead pull down

mEphA4^LBD^ was immobilized on protein G beads to pull down zefn-A5^ECD^ or the zefn-A5^ECD^/zRET^ECD^ complex. Samples were prepared as described for the anti-Flag pull down assay but replacing anti-Flag resin with protein G agarose beads (Pierce, Thermo Fisher Scientific). Washed beads were incubated with SDS-loading dye and were subjected to WB detection with 4–20% Coomassie-stained SDS-PAGE. Experiments were performed in triplicate.

## Results

We used native gel electrophoresis, mass spectrometry, pull down assays, and quantitative binding studies to try identify a clear biochemical interaction between zRET^ECD^ and zefn-A5^ECD^ corresponding to that previously identified in cell-based assays [13].

### Native Gel electrophoresis and MS analysis

**Fig 1:**
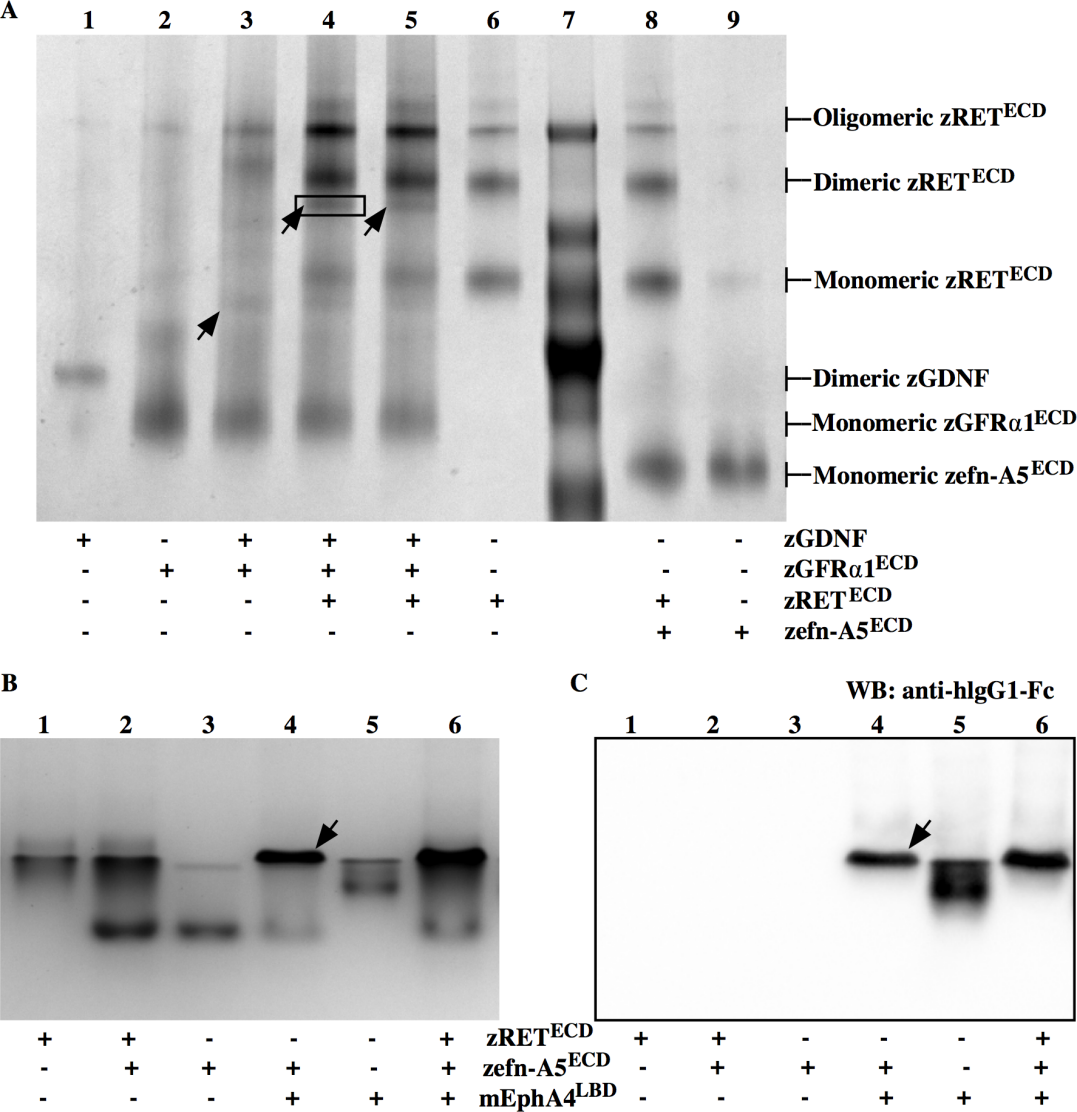
Coomassie-stained Native PAGE of zGDNF/zGFRα1^ECD^/zRET^ECD^ and mEphA4^LBD^/zefn-A5^ECD^ complexes. (A) BN PAGE image of zGDNF/zGFRα1^ECD^/zRET^ECD^ complex. A molar ratio of 1:1:1 based on the monomeric forms of the proteins was used for zGDNF, zGFRα1^ECD^ and zRET^ECD^. zRET^ECD^ and zefn-A5^ECD^ were incubated with a molar ratio of 1:2. The formation of zGDNF_2_/zGFRα1_2_^ECD^ complex was shown in Lane 3 (black arrow). The band corresponding to the ternary zGDNF_2_/zGFRα1^ECD^/zRET^ECD^ complex is shown in Lanes 4 and 5 (black arrows). Lanes 4 and 5 are duplicates. The band highlighted with solid rectangle (Lane 4) was analyzed using mass spectrometry (S1 Table). (B) Clear Native (CN) PAGE of the mEphA4^LBD^/zefn-A5^ECD^ complex. mEphA4^LBD^/zefn-A5^ECD^ were incubated with a molar ratio of 1:2. The formation of mEphA4^LBD^/zefn-A5^ECD^ complex is marked with a black arrow. (C) Anti-hlgG1-Fc Western blotting showing the mEphA4^LBD^/zefn-A5^ECD^ complex formation in Lane 4 (black arrow).

Blue Native PAGE has been widely used to visualize qualitatively the formation of protein complexes in their native condition [31]. Since zGDNF and zGFRα1^ECD^ have been previously shown to interact with each other in the absence of zRET^ECD^ [32,33], we choose this complex and the zGDNF/zGFRα1^ECD^/zRET^ECD^ tripartite complex as positive controls for our experiments. In accordance with previous studies, we observed the “binary” complex of expected stoichiometry, GDNF_2_/GFRα1_2_ (Fig 1A, black arrow in Lane 3). Furthermore, after the addition of zRET^ECD^, zGDNF_2_/zGFRα1^ECD^/zRET^ECD^ associated with each other with an estimated stoichiometry of 2:1:1 shown as the bands below the band of zRET^ECD^ dimer (Fig 1A, black arrows in Lanes 4 and 5). The complex formation was further verified by mass spectrometry (S1 Table). Because the stoichiometry of the potential zRET^ECD^/zefn-A5^ECD^ complex remained unclear, we incubated zRET^ECD^ and zefn-A5^ECD^ together with a molar ratio of 1:2. Contrary to our hypothesis, no apparent complex formation was observed when zRET^ECD^ and zefn-A5^ECD^ were incubated together in the absence of zGDNF. This was shown by comparing the Coomassie-stained bands in Lane 8 to those in Lane 6 and 9 (Fig 1A). Same observation was made also in the presence of both zGDNF and zGFRα1^ECD^ (S2 Fig).

Next, we examined the complex formation between zRET^ECD^ and zefn-A5^ECD^ by Clear Native (CN) PAGE. There are no extra bands in Fig 1B, Lane 2 in comparison with those in Lane 1 and Lane 3. Additionally, the band intensity for zefn-A5^ECD^ in Lane 2 remained the same as the one in Lane 3 (measured by ImageJ, data not shown), which indicates that no or a non-observable amount of zefn-A5^ECD^ formed a complex with zRET^ECD^. Given the high sequence similarity among efn-A5 orthologs (S1 Fig) and the strong 1:1 interaction between EphA4 and efn-A5 [34], we tested whether zefn-A5 forms a complex with mEphA4^LBD^. The mEphA4^LBD^/zefn-A5^ECD^ complex formation could not be visualized by BN PAGE (S3 Fig), but was detected on a CN PAGE gel (Fig 1B, black arrow). The latter results were further verified by western blotting (Fig 1C). Because mEphA4^LBD^ and zefn-A5^ECD^ were incubated with a molar ratio of 1:2 to be consistent with the ratio of zRET^ECD^ and zefn-A5^ECD^ used in this experiment as described previously, all mEphA4^LBD^ formed a complex with zefn-A5^ECD^, while one third of the zefn-A5^ECD^ did not (Fig 1B**,** Lanes 3 and 4) as measured by ImageJ (data not shown). The unbound zefn-A5 was less than half of the amount of zefn-A5 added, which may be a result of dimerization of some of the protein upon receptor binding. Additionally, adding zRET^ECD^ to a mixture of mEphA4^LBD^ and zefn-A5^ECD^ did not have an effect on complex formation (Fig 1B and 1C, both Lanes 6).

### Pull-down assays

**Fig 2:**
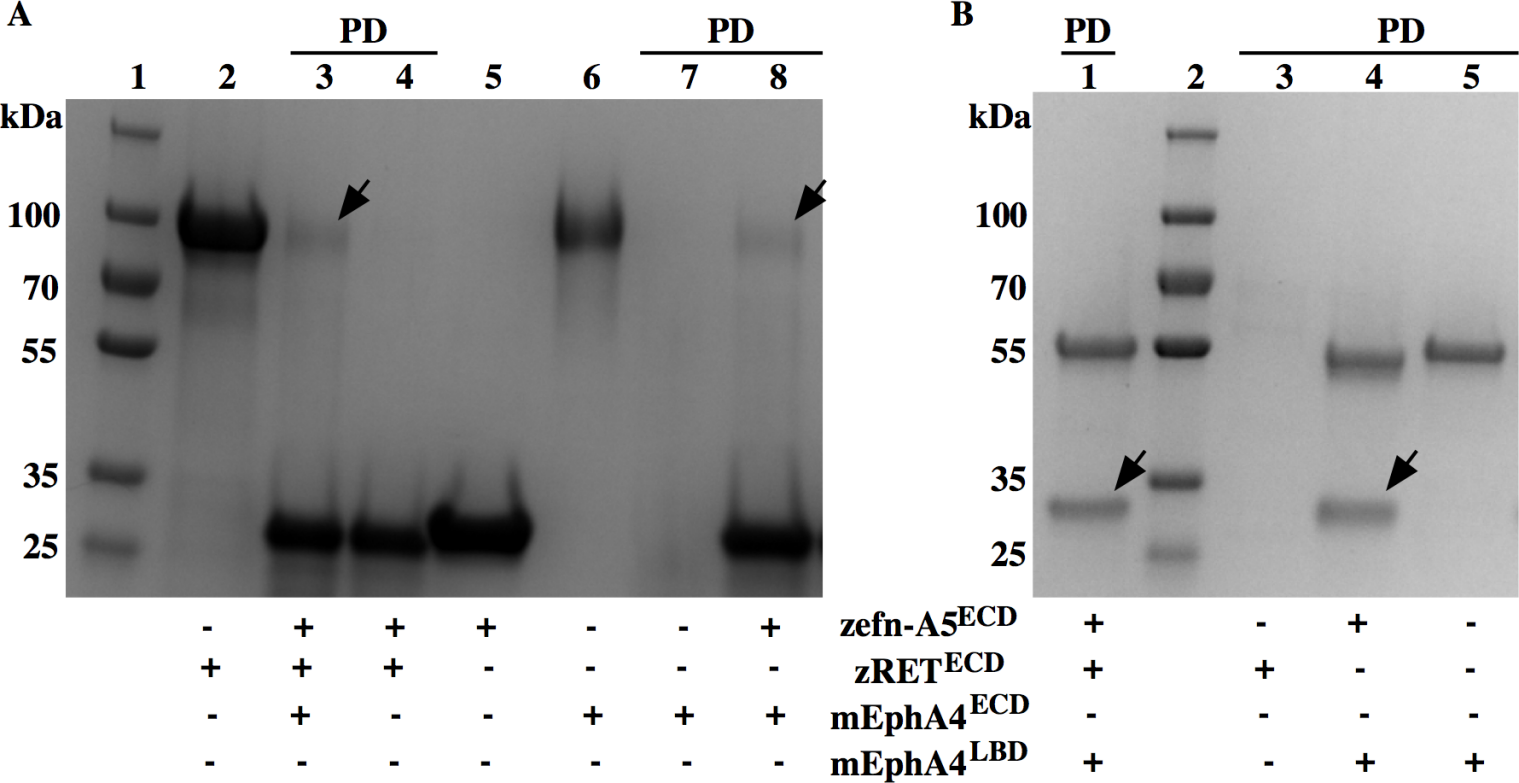
Pull-down assays of zefn-A5^ECD^, mEphA4^ECD^, mEphA4^LBD^ and zRET^ECD^. (A) Anti-Flag resin pull down of mEphA4^LBD^ and untagged zRET^ECD^ with immobilized zefn-A5^ECD^. The black arrows in Lanes 3 and 8 point to the mEphA4^LBD^ pulled down by zefn-A5^ECD^. (B) Protein G bead pull down of zRET^ECD^ and zefn-A5^ECD^ with immobilized mEphA4^LBD^. The black arrows in Lanes 1 and 4 mark the zefn-A5^ECD^ pulled-down by mEphA4. Molecular weight standards: PageRuler Plus standard protein ladder in Lanes 1(A) and 2(B). PD: samples eluted from the beads after pull down. Lanes without the PD label contain input protein samples as indicated.

We also tried to see if zRET^ECD^ interacted with zefn-A5^ECD^ using pull-down experiments. As a positive control, we used zefn-A5^ECD^ to pull down mEphA4^LBD^ (Fig 2A, Lanes 3 and 8) and *vice versa* (Fig 2B, Lanes 1 and 4). zefn-A5^ECD^ runs at approximately 30 kDa and the pulled down mEphA4^ECD^ runs at 100 kDa as shown in Fig 2A (Lanes 3 and 8). This verified that the purified zefn-A5^ECD^ was active. In agreement with the other experiments presented here, untagged zRET^ECD^ was not pulled down by zefn-A5^ECD^. According to Bonanomi *et al*. [13], the presence of EphAs and GDNF facilitates the association between zefn-A5^ECD^ and zRET^ECD^ *in cellulo* by mediating the correct localization and clustering of the two proteins to lipid rafts. To investigate whether EphA4 could bridge the interaction between zefn-A5^ECD^ and zRET^ECD^ *in vitro*, we used mEphA4^LBD^ immobilized on protein G beads to pull down pre-incubated of zefn-A5^ECD^ and zRET^ECD^. However, the results were consistent with the anti-Flag pull down and the native PAGE results: zRET^ECD^ was pulled-down neither by zefn-A5^ECD^ nor by mEphA4^LBD^ together with zefn-A5^ECD^ (Fig 2B, Lanes 1 and 4).

### Quantitative binding assays

**Fig 3:**
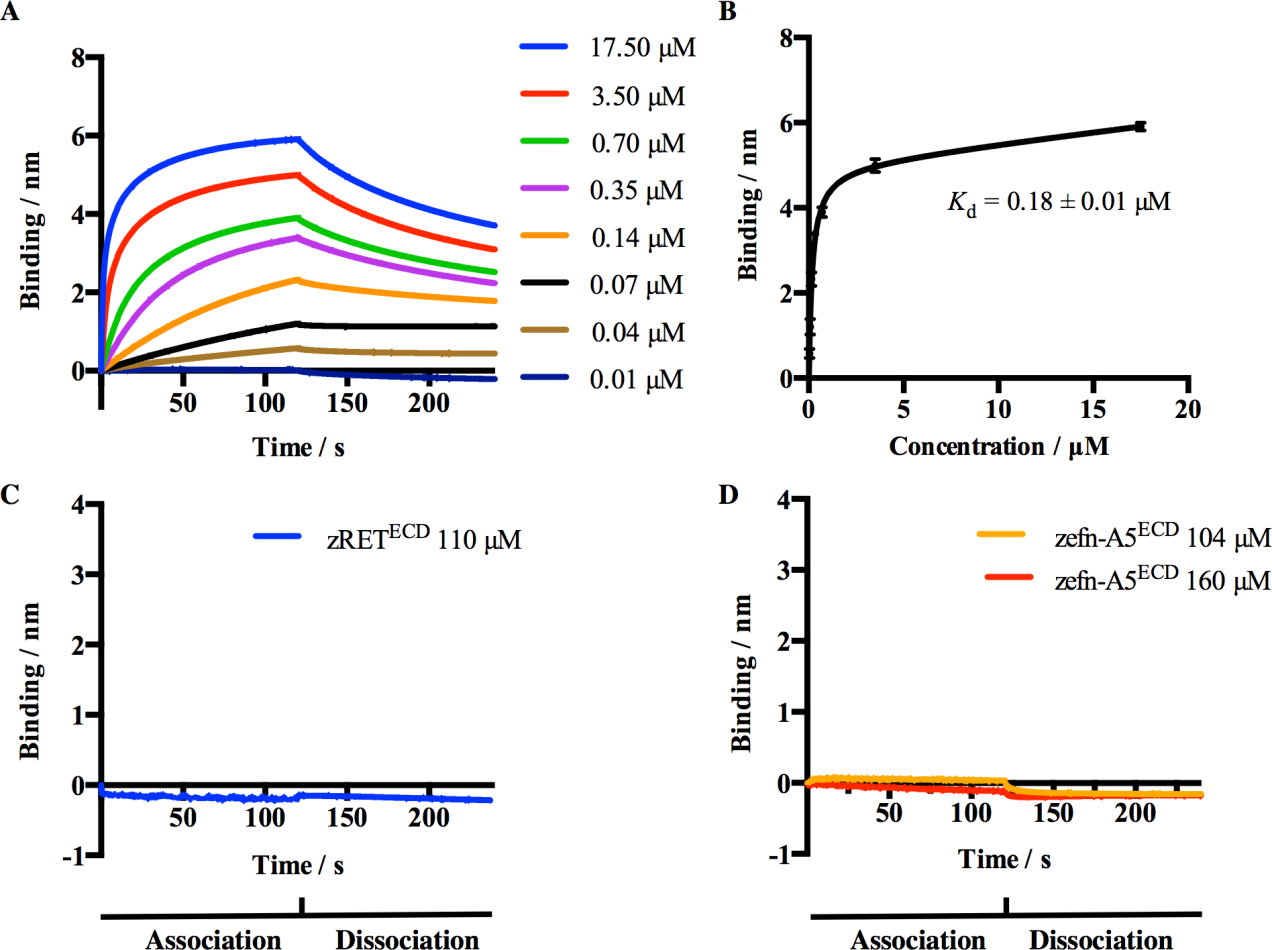
Measurement of the affinity of zefn-A5^ECD^ for mEphA4^LBD^ and zRET^ECD^. (A) BLItz sensorgrams showing mEphA4^LBD^ binding to immobilized zefn-A5^ECD^ at the indicated concentrations. (B) Saturation binding curve fitted with 1:1 binding model, using GraphPad Prism 6, showing the binding of mEphA4^LBD^ to immobilized zefn-A5^ECD^. The binding model allowed non-specific binding, which occurs as shown by the fact that the baseline is not horizontal. The x-axis represents the concentration of mEphA4^LBD^. (C) Sensorgram showing untagged zRET^ECD^ binding to immobilized zefn-A5^ECD^ at a concentration of 110 μM. The binding profile was derived from three independent experiments and plotted with mean value against running time. (D) Sensorgram showing untagged zefn-A5^ECD^ binding to immobilized zRET^ECD^ at two concentrations of 104 and 160 μM.

Finally, we used bio-layer interferometry technology system (BLItz) to measure the binding affinity and kinetics between zRET^ECD^ and zefn-A5^ECD^. As a positive control, we collected sensorgrams for mEphA4^LBD^ at eight different concentrations binding to immobilized zefn-A5^ECD^ on a Ni-NTA biosensor and then dissociating from the surface by dipping the biosensor into buffer (Fig 3A). The binding response reported at saturation is shown as a function of mEphA4^LBD^ concentration in Fig 3B, fitted with 1:1 binding model. As expected, our results show that mEphA4^LBD^ exhibited high affinity for monomeric zefn-A5^ECD^ with a dissociation constant (*K*_d_) of 0.18 ± 0.01 μM (Fig 3A and 3B). To determine if there was binding between zRET^ECD^ and zefn-A5^ECD^, we collected sensorgrams not only for untagged zRET^ECD^ binding to immobilized zefn-A5^ECD^ (Fig 3C) but also *vice versa* (Fig 3D). In accordance with our previous results, neither showed detectable binding affinity towards each other.

## Discussion

### Functional proteins produced in insect cells

We used baculovirus expression vector system (BEVS) to express all the proteins for the assays described above. To verify that the proteins were in their expected oligomeric state and functional, a series of experiments were conducted. Analysis of zRET^ECD^ using native PAGE showed that secreted zRET^ECD^ exists mainly as a mixture of monomer and dimer with a small portion of higher oligomers (Fig 1A). zRET^ECD^ formed the expected complex with zGDNF/zGFRα1^ECD^, confirming its functionality [24]. zefn-A5^ECD^ produced by BEVS is monomeric in solution as shown by native PAGE results (Fig 1), consistent with the results observed for hefn-A5 expressed in mammalian cells [35,36] as well as for efn-A1 expressed by insect cells [37]. To see that the produced zefn-A5^ECD^ is functional and thus capable of binding mEphA4^LBD^, we conducted measurements using BLItz. As expected, monomeric zefn-A5^ECD^ bound mEphA4^LBD^ with submicromolar affinity (*K*_d_ = 0.18 ± 0.01 μM, Fig 3B). The affinity is 20 times weaker than that between mefn-A5^ECD^ and mEphA3-Fc (*K*_d_ = 0.008 μM) [38], but essentially the same as that between hefn-A5^ECD^ (also referred as the receptor-binding domain of hefn-A5, hefn-A5^RBD^) and hEphA4^LBD^ (*K*_d_ = 0.36 μM) [34] measured by surface plasmon resonance, suggesting that the binding interfaces of efn-A5 orthologs are highly conserved.

### Blue native PAGE may affect complex formation

Based on our results, a complex of mEphA4^LBD^ and zefn-A5^ECD^ was observed in clear native (CN) (Fig 1) but not in blue native (BN) PAGE (S3 Fig). In BN PAGE, Coomassie dye is bound to the protein in the sample buffer, and provides a single negative charge per dye molecule bound. As a result, the protein-dye complex overall has negative charge. Unlike BN PAGE, CN PAGE uses Ponceau dye in the sample buffer and the dye does not bind to protein under the running condition: proteins with a pI below the pH of the running buffer migrate into the gel with a conventional electrode set up and the mobility depends not only on the molecular size and shape but, to a large extent, on their intrinsic charge [39,40]. Formation of the mEphA4^LBD^/zefn-A5^ECD^ complex was seen in CN PAGE but not BN PAGE. Why could this be so? Both mEphA4^LBD^ and zefn-A5^ECD^ migrated in BN PAGE when they were incubated together (S3 Fig, Lane 6) and the intensity of the bands did not change compared to that when they were run separately (S3 Fig, Lanes 2 and 3). This suggests that Coomassie dye may have disrupted the complex formation between mEphA4^LBD^ and zefn-A5^ECD^ due to its protein-binding ability and the electrical repulsion the dye creates between protein-binding interfaces. We nonetheless believe that this does not explain the absence of the zRET^ECD^/zefn-A5^ECD^ complex. Both proteins migrate into the CN and BN PAGE when loaded separately or together (Fig 1), and so potential Coomassie-dye induced interference of the binding between zRET^ECD^ and zefn-A5^ECD^ is not relevant.

### zefn-A5^ECD^ and zRET^ECD^ do not interact directly *in vitro*

We observed that zefn-A5^ECD^ and zRET^ECD^ did not interact directly with each other. Immunoprecipitation and neuron-based studies [13] suggested that mefn-A5^ECD^ competes with mGFRα1 *in cellulo* to bind to mRET9, and that mGDNF may mediate the binding between mRET^ECD^ and mefn-A5^ECD^ by directing them into lipid rafts. Similarly, Chai *et al*. [14] reported that mRET, mefn-A and mGFRα1 might form a protein complex meditating efn-A reverse signaling together with Celsr3/Fzd3. Therefore, we also tested if zRET^ECD^ could bind to zefn-A5^ECD^ in the presence of zGDNF and zGFRα1^ECD^ *in vitro*. However, BN PAGE showed that neither zGDNF/zefn-A5^ECD^/zRET^ECD^ nor zGDNF/zGFRα1^ECD^/zefn-A5^ECD^/zRET^ECD^ formed a complex when the proteins were incubated together, and the same result was observed for zefn-A2^ECD^ in lieu of zefn-A5^ECD^ (S2 Fig). Therefore, our results are consistent with GDNF indirectly mediating zefn-A5/zRET association [13] *in cellulo*.

**Fig 4:**
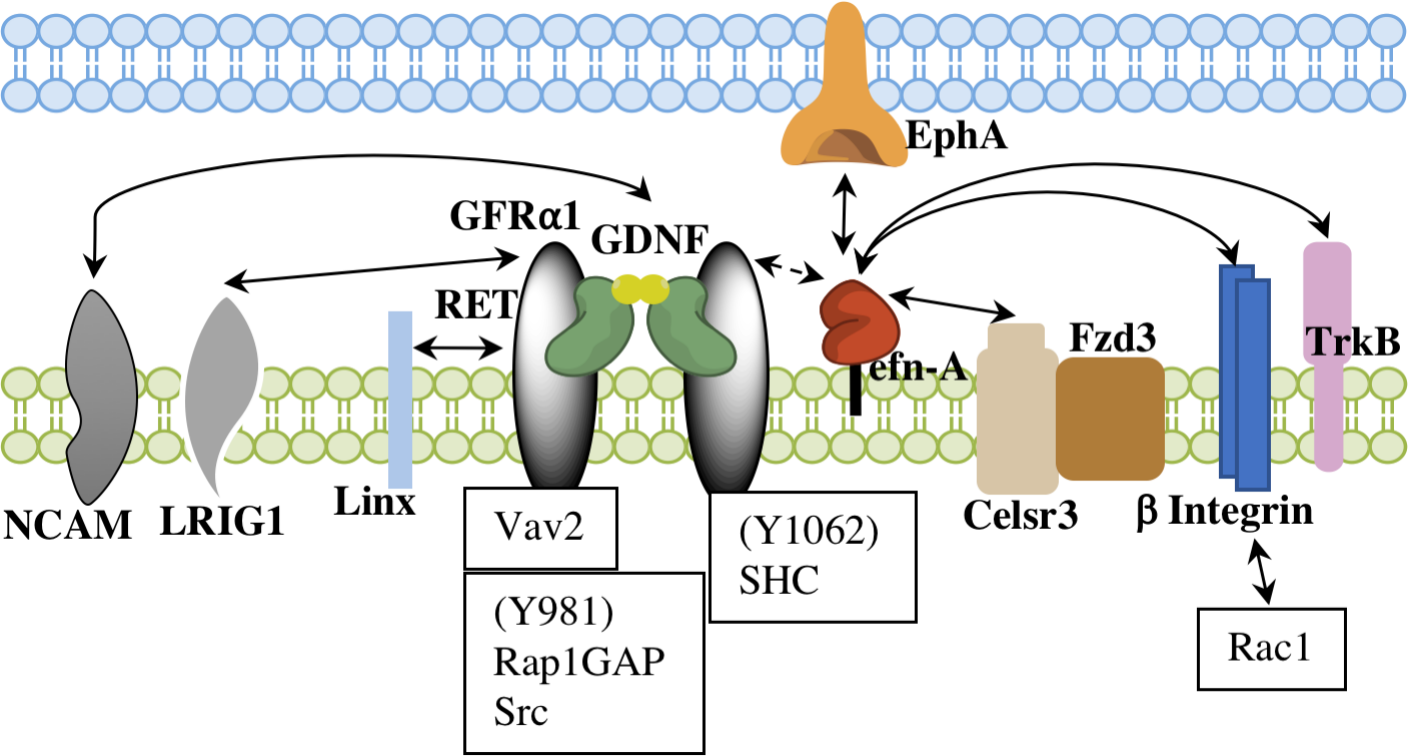
Schematic model of RET signaling. EphrinAs, GFRα1, GDNF and RET may interact together with Celsr3/Fzd3 [14] to transduce efn-As reverse signaling. β integrins [9] as well as leucine-rich repeat and immunoglobulin (LRIG) family proteins, such as LRIG1 [48] and Linx [49], may play a role in RET-mediated efn-As signaling. NCAM [50,51]: neural cell adhesion molecule; LRIG1 [48,52]: leucine-rich repeats and immunoglobulin-like domains; TrkB: tropomyosin receptor kinase B.

Saarenpää *et al*. [24] reported that hRET could be stimulated upon the binding of zGFRα1^ECD^/zGDNF, implying the structural conservation of the GFRα1/GDNF binding domain of RET. We therefore hypothesized that the proposed RET-efn-A5 interaction in mouse [13,14] should also be conserved in zebrafish and efn-A5 and GFRα1 shared the same or occupied an adjacent binding site. Therefore, in this study, we examined the interaction between zRET^ECD^ and zefn-A5^ECD^. On contrary to our hypothesis, no interaction was observed between purified zefn-A5^ECD^ and zRET^ECD^. One potential explanation for the lack of association between efn-A5 and RET is that another binding partner that is present only in the cell-based assays mediates their interaction. As mentioned earlier, reverse signaling of mEphAs/mefn-As through mRET takes place on the cell surface, where mEphAs and mefn-As interact *in trans* and where Bonanomi *et al*. [13] suggest that mefn-As and mRET associate with each other *in cis* (Fig 4). Their results demonstrated that mefn-As and mRET are colocalized on the cell surface and that mRET-involved mefn-As reverse signaling is responsible for correct dorsal projection of motor axon and dendrite outgrowth stimulated by GDNF and mEphA7 [13,14,17]. However, these results do not demonstrate direct interaction between efn-A5 and RET; had there been one, it should have been detectable *in vitro* with two purified proteins. The association between zefn-A5^ECD^ and zRET^ECD^ could not be measured in our study even at high concentrations (Fig 3). It strongly suggests that the zefn-A5-zRET is indirect.

Previous investigations have suggested that there are a number of efn-A5 binders including Celsr3/Fzd3 [14], TrkB [10] and p75 neurotrophin receptor (p75NTR) on retinal ganglion cells [11,41]. Using transfected mammalian cells, mRET, mGFRα1 and mefn-As were shown to be co-immunoprecipitated by Celsr3 and Fzd3 and the role Celsr3/Fzd3 played was found to be specific for efn-A reverse signaling [14]. Based on our results, purified zRET^ECD^, zGFRα1^ECD^ and zefn-A^ECD^ do not form a complex in the presence or absence of zGDNF. However, it would still be interesting to examine the complex formation *in vitro* with purified zebrafish Celsr3 and Fzd3. Furthermore, Marler *et al*. [10] reported efn-As interacted with TrkB for their reverse signaling by binding with the cysteine-rich domain 2 (CC2) of TrkB, although the interaction between the extracellular domains of efn-A2-Fc and TrkB-Fc was not detected by SPR [42]. It would be interesting to see if there is another binding partner involved in the interaction of TrkB with efn-As, which could explain the lack of interaction in the *in vitro* studies between efn-A2-Fc and TrkB-Fc, similar to our observation with zRET^ECD^ and zefn-A5^ECD^. Soba *et al*. reported direct interaction between *Drosophila melanogaster* RET and integrins [43]. As another binding partner of efn-As, β1 integrins cooperate with efn-As *in cis* in the presence of EphA, transducing reverse signaling of efn-A [9]. The ensemble of complex signaling cues suggest that the receptors might interact directly, where cell-cell communication occurs, to integrate the multiple cues and transduce the repulsive or attractive signals to downstream pathways. More recently, Mullican *et al*. [44], Yang *et al*. [45], Emmerson *et al*. [46] and Hsu *et al*. [47] reported another ligand pair for RET, growth differentiation factor 15 (GDF15) and GDNF family receptor α-like (GRAL). Being a member of the transforming growth factor β (TGF-β) superfamily, GDF15 selectively bound to GRAL, rather than GFRαs, with high affinity. The findings revealed the complexity of RET-mediated signaling, suggesting that another, as yet unidentified ligand, might also be involved in efn-A/RET signaling. It will therefore be interesting to investigate whether Celsr3/Fzd3, integrins or other unidentified proteins play a role in the reverse signaling of efn-A5 with RET *in vitro*.

## Supporting information

S1 Fig Amino acid sequence alignment of zebrafish, human and mouse efn-A5b or efn-A5 isoforms using T-Coffee. Grey and black shades are to show conserved and identical amino acids, respectively. Signal sequence is excluded for all inputs.
(TIF)

S2 Fig Coomassie-stained Blue Native PAGE images of zRET^ECD^, zefn-A2^ECD^, zefn-A5^ECD^, zGFRα1^ECD^ and zGDNF. (A) No complex formation was observed when zefn-A5^ECD^ was added to mixtures of zRET^ECD^/zGFRα1^ECD^/zGDNF (Lane 5) or zRET^ECD^/zGFRα1^ECD^ (Lane 9). zGDNF_2_/zGFRα1_2_^ECD^ complex was shown in Lane 3 (black arrow). Ternary zGDNF_2_/zGFRα1^ECD^/zRET^ECD^ complex is shown in Lanes 4 and 5 (black arrows). (B) No complex formation was observed when zRET^ECD^/zefn-A5^ECD^/zGDNF (Lane 1), zefn-A5^ECD^/zGDNF (Lane 3) were incubated together. (C) No complex formation was observed when zRET^ECD^/zGDNF (Lane 3), or zRET^ECD^/zefn-A2^ECD^/zGDNF (Lane 4) were incubated together.
(TIF)

S3 Fig Coomassie-stained Blue Native PAGE images of zRET^ECD^, mEphA4^LBD^ and zefn-A5^ECD^. No complex formation was observed when zRET^ECD^/zefn-A5^ECD^ (Lane 4), zefn-A5^ECD^/mEphA4^LBD^ (Lane 7) or zRET^ECD^/mEphA4^LBD^ (Lane 5) were incubated together.
(TIF)

S1 Table Proteins identified in gel slice from Blue Native PAGE using LC-MS/MS. Gel slice contains the band in Fig 1A (solid rectangle).
(XLSX)
