# Supplementary figures and images for: Binding of EphrinA5 to RET receptor tyrosine kinase: An in vitro study
Source: PLoS One. 2018 Jun 11;13(6):e0198291. doi: 10.1371/journal.pone.0198291 (PMC5995387; doi:10.1371/journal.pone.0198291)

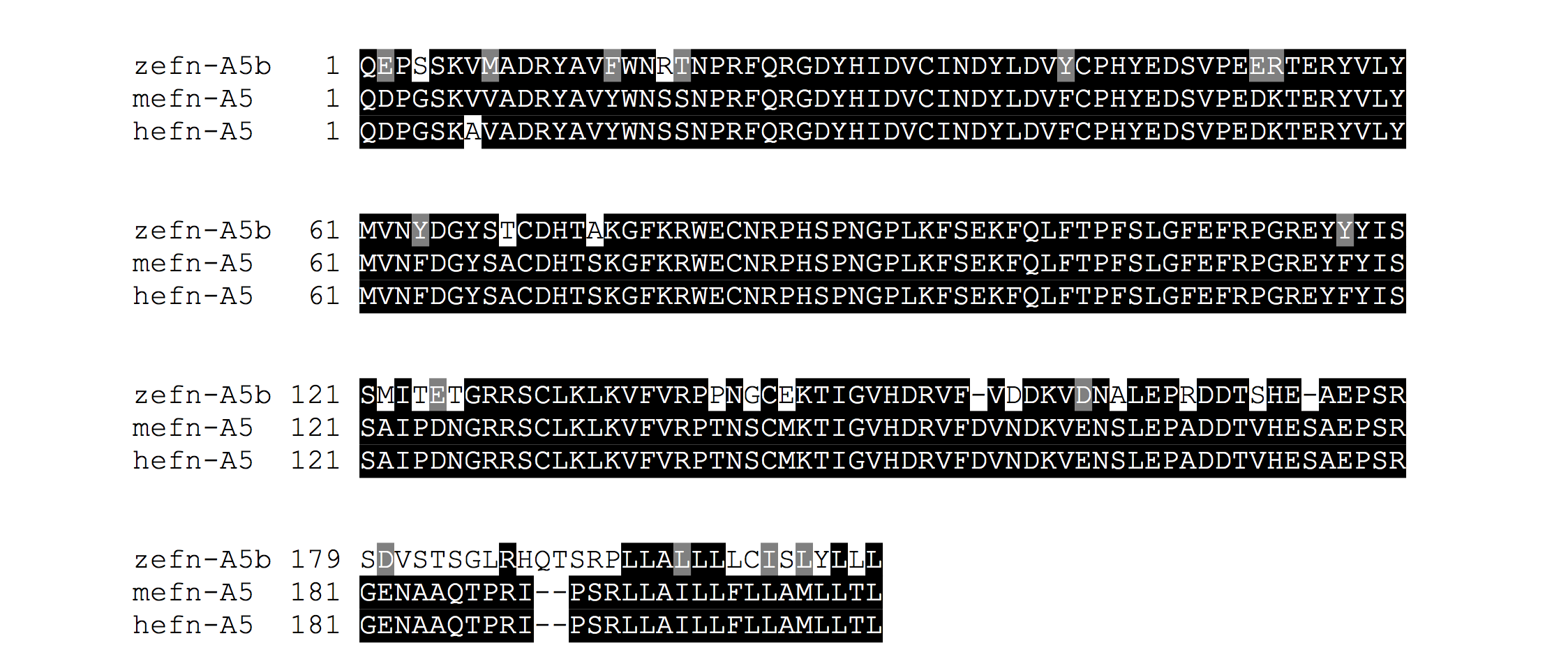

Supplement: S1 Fig — Grey and black shades are to show conserved and identical amino acids, respectively. Signal sequence is excluded for all inputs. (TIF) [file pone.0198291.s001.tif]

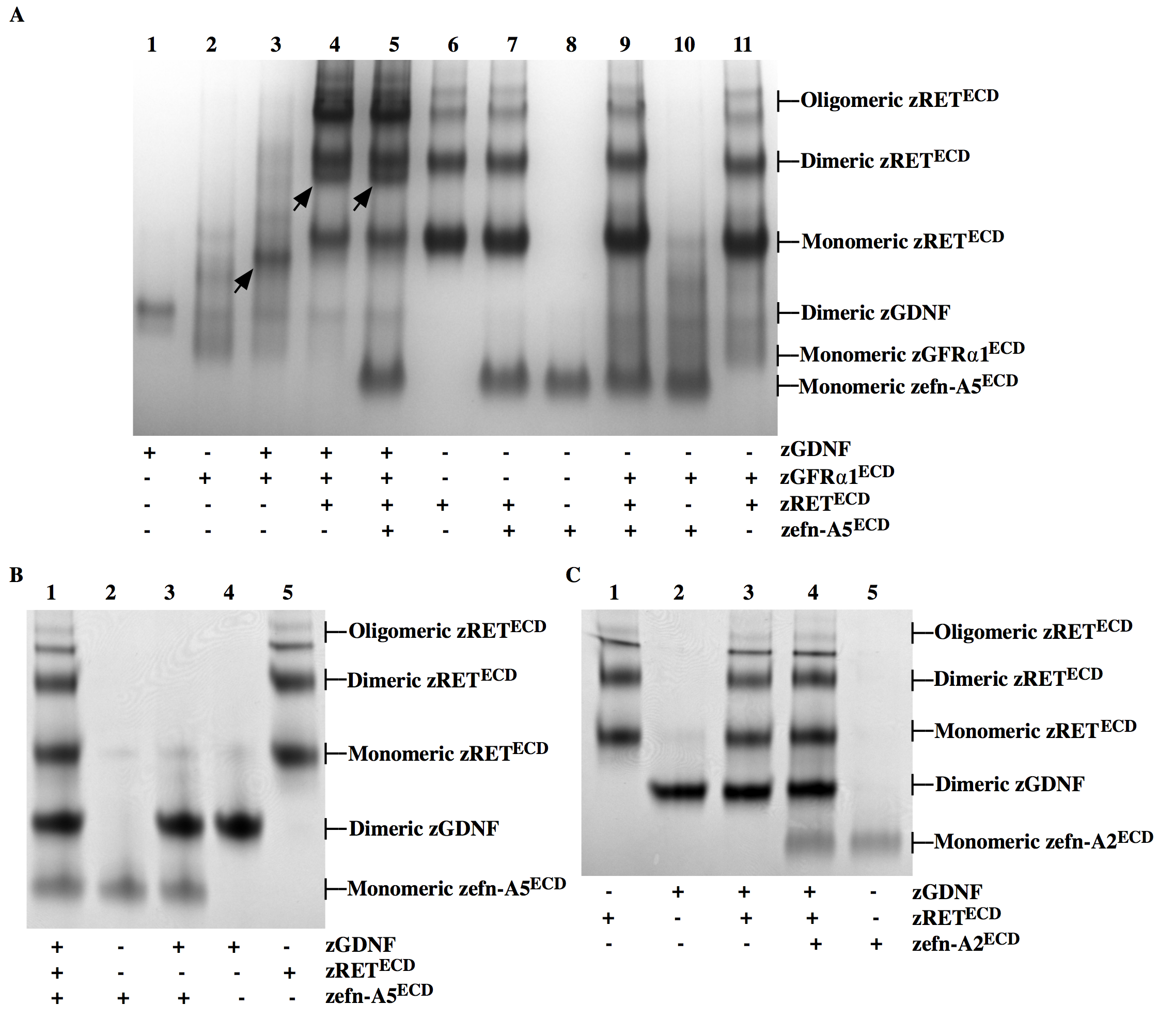

Supplement: S2 Fig — (A) No complex formation was observed when zefn-A5ECD was added to mixtures of zRETECD/zGFRα1ECD/zGDNF (Lane 5) or zRETECD/zGFRα1ECD (Lane 9). zGDNF2/zGFRα12ECD complex was shown in Lane 3 (black arrow). Ternary zGDNF2/zGFRα1ECD/zRETECD complex is shown in Lanes 4 and 5 (black arrows). (B) No complex formation was observed when zRETECD/zefn-A5ECD/zGDNF (Lane 1), zefn-A5ECD/zGDNF (Lane 3) were incubated together. (C) No complex formation was observed when zRETECD/zGDNF (Lane 3), or zRETECD/zefn-A2ECD/zGDNF (Lane 4) were incubated together. (TIF) [file pone.0198291.s002.tif]

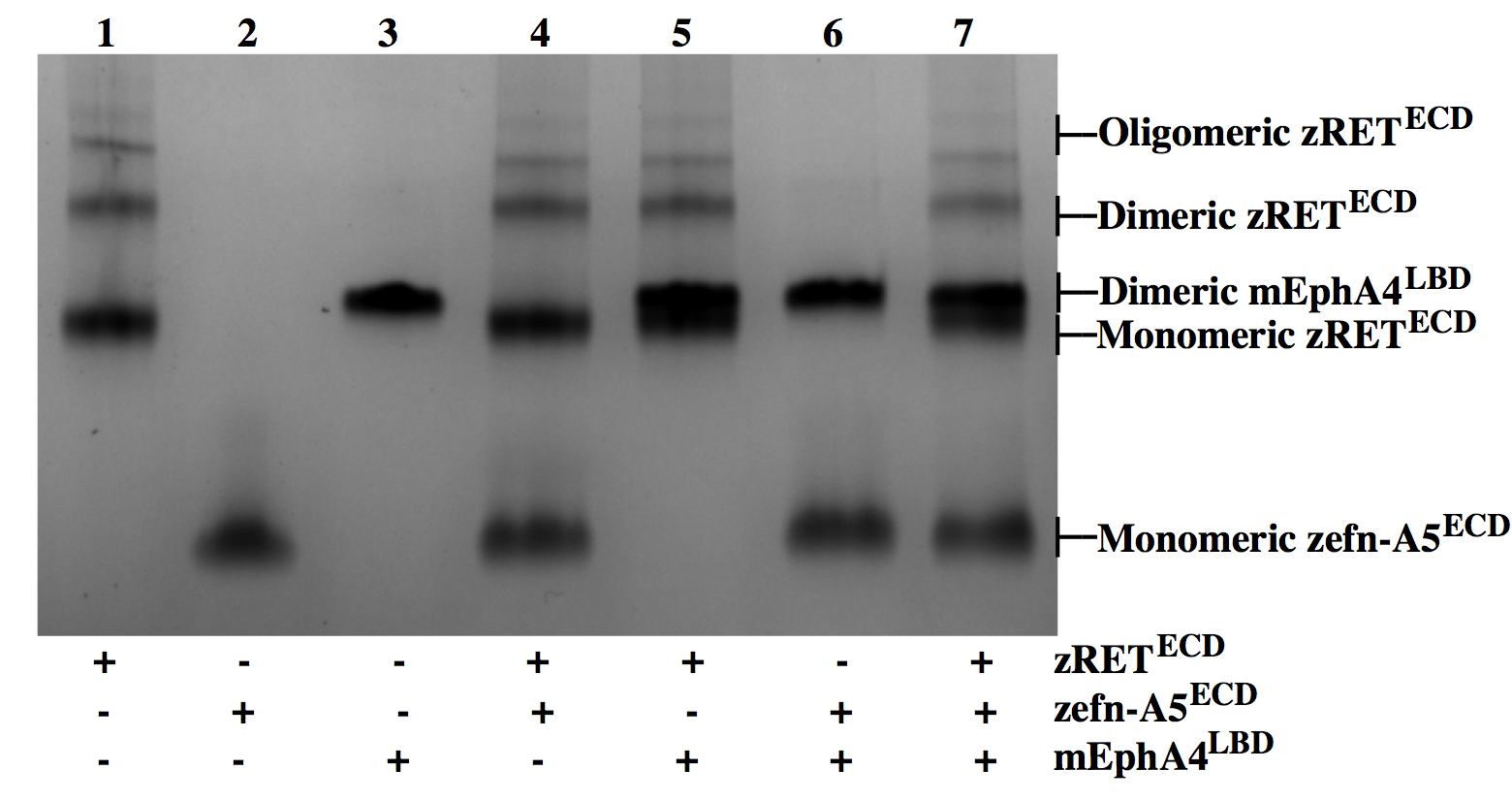

Supplement: S3 Fig — No complex formation was observed when zRETECD/zefn-A5ECD (Lane 4), zefn-A5ECD/mEphA4LBD (Lane 7) or zRETECD/mEphA4LBD (Lane 5) were incubated together. (TIF) [file pone.0198291.s003.tif]
